# Supplementary material for: Identification of photoperiod-induced specific miRNAs in the adrenal glands of Sunite sheep (Ovis aries)
Source: Front Vet Sci. 2022 Jul 22;9:888207. doi: 10.3389/fvets.2022.888207 (PMC9354845; doi:10.3389/fvets.2022.888207)
Supplement: Supplementary file 1 [file Data_Sheet_1.ZIP › Supplementary file/miRDeep2 file.zip!/miRDeep2 file/Know_Structure/SPLP42A2/oar-mir-329b.pdf]

miRBase precursor : oar-mir-329b
Total read count : 2542
oar-miR-329b-5p read count : 22
oar-miR-329b-3p read count : 2495
remaining reads : 25

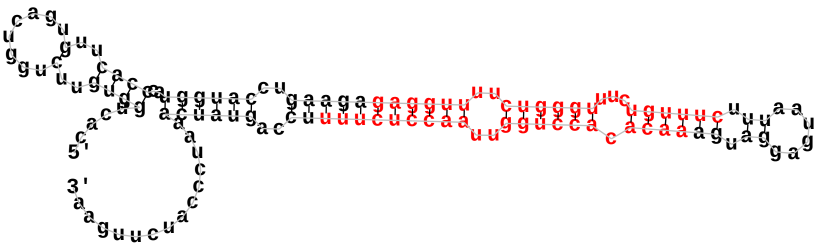

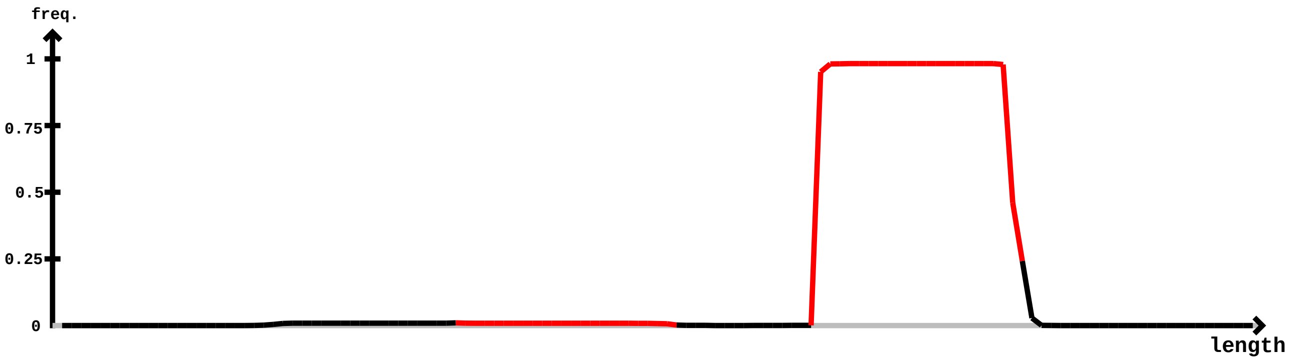


```
                                                                           oar-miR-329b-3p
                                          oar-miR-329b-5p
5'- cacugguguucuggucaguguucacccaugguaccugaagagagguuuucuggguuucuguuucuuuaaugaggaugaaacacaccugguuaaccucuuuuccaguaucaaaucccaucuugaa  -3'   exp
    ....((((..(........)..))))..((((((..((((((((((..((((((...((((((.((......)).)))))).))))))..))))))))))..))))))................  reads     mm
    ...................guucacccaugguaccugaaga...................................................................................  1         0
    ....................uucacccaugguaccugaaga...................................................................................  3         0
    .....................ucacccaugguaccugaag....................................................................................  1         0
    .....................ucacccaugguaccugaaga...................................................................................  6         0
    ......................cacccaugguaccugaaga...................................................................................  9         0
    .......................acccaugguaccugaaga...................................................................................  2         0
    .......................acccaugguaccugaagag..................................................................................  1         0
    ........................................agagguuuucuggguuucugu...............................................................  1         0
    ........................................agagguuuucuggguuucuguu..............................................................  2         0
    ........................................agagguuuucuggguuucuguuu.............................................................  1         0
    .........................................gagguuuucuggguuucu.................................................................  1         0
    .........................................gagguuuucuggguuucugu...............................................................  1         0
    .........................................gagguuuucuggguuucuguuu.............................................................  11        0
    .........................................gagguuuucuggguuucuguuuc............................................................  3         0
    .........................................gagguuuucuggguuucuguuucuuu.........................................................  2         0
    .......................................................................aggaugaaacacaccugguuaaccucu..........................  1         0
    ...........................................................................ugaaacacaccugguuaaccucu..........................  1         0
    ..............................................................................aacacaccugguuaaccuc...........................  9         0
    ..............................................................................aacacaccugguuaaccucu..........................  1311      0
    ..............................................................................aacacaccugguuaaccucuu.........................  551       0
    ..............................................................................aacacaccugguuaaccucuuu........................  523       0
    ..............................................................................aacacaccugguuaaccucuuuu.......................  22        0
    ...............................................................................acacaccugguuaaccucu..........................  5         0
    ...............................................................................acacaccugguuaaccucuu.........................  4         0
    ...............................................................................acacaccugguuaaccucuuu........................  20        0
    ...............................................................................acacaccugguuaaccucuuuu.......................  47        0
    ................................................................................cacaccugguuaaccucuuuu.......................  1         0
    .................................................................................acaccugguuaaccucuuuuc......................  1         0
    .................................................................................acaccugguuaaccucuuuucc.....................  1         0
```
